# Surgical Homes

**Published:** 1906-06-02

**Authors:** 


### SURGICAL HOMES.

### HOW NOT TO DO IT.

An appeal has been sent to us on behalf of a so-called home hospital for £2,500 for a building fund. The utter want of apprehension of plain facts which zealous enthusiasts, with a sincere desire to ride a hobby hard, may exhibit, is proved by some reasons for rebuilding which accompany this appeal. It appears from these that the Home in question was not in a sanitary condition, and that when it was made safe in this respect the site was so restricted as to make the insanitation of neighbouring buildings a danger to the inmates. The space around the house is so limited that the only position where the patients can be placed in the open air affords little shade in summer and is exposed in the winter. There is no isolation accommodation except an attic boxroom. "The possibility of escape in case of fire has puzzled the experts"; the only staircase is an awkward one, although all the inmates are helpless except one, and have to be carried up and down. There is only one bathroom for the use of the staff and the patients. The two nurses occupy the same bedroom, have no sitting-room, and take their meals in the day-room, for want of other accommodation.

To the ordinary business mind such facts as these would have forbidden any attempt to use such accommodation for the treatment of patients at all. Having taken these risks and submitted the patients to the disadvantages referred to, it is evidence of a somewhat curious state of mind that the managers should now put forth these disabilities as the main basis of an appeal for £2,500. In our view institutions started under such conditions are a mistake, and we must strongly condemn the promoters of such enterprises, who merely minister to the degeneracy of many fashionable people who take up charities as a fad, and have so little heart and mind in this work that, as has been recorded, one lady hurrying off to a meeting recently was unable to say whether she was going to support an institution for dogs or for children! We would caution those who have money to give in charity to discourage ill-considered novelties, where the promoters show a total absence of apprehension of the responsibilities which should attach to every person who puts herself or himself at the head of a movement, which must depend upon voluntary contributions for its maintenance and progress. In practice institutions started like the one here referred to create at the outset an evil, and, having done so, the promoters make its existence the basis of a scheme for contributions to enable it to be remedied. How can the authors of such proceedings expect to win public confidence for their work?

